# Seroprevalence of Anti-SARS-CoV-2 IgG Antibodies among Children attending the Pediatric Hospital in Bamako, Mali (BamaCoV-Kids Study)

**DOI:** 10.21203/rs.3.rs-4983012/v1

**Published:** 2024-09-26

**Authors:** Almoustapha Issiaka Maiga, Amadou Kodio, Salimata Alou Ouedrago, Aliou Baldé, Penda Dembele, Fatoumata Tata Traore, Oumar Dolo, Josué Togo, Yacouba Aba Coulibaly, Mariam Sylla, Robert L. Murphy, Anne-Geneviève Marcelin, Vincent Calvez, Abdoul Aziz Diakité, Eve Todesco

**Affiliations:** Centre Hospitalier Universitaire Gabriel Touré; Unité d’Epidémiologie Moléculaire de la Résistance du VIH aux ARV, University of Sciences, Techniques and Technologies of Bamako; Unité d’Epidémiologie Moléculaire de la Résistance du VIH aux ARV, University of Sciences, Techniques and Technologies of Bamako; Sorbonne Université, INSERM, Institut Pierre Louis d’Épidémiologie et de Santé publique (iPLESP); Unité d’Epidémiologie Moléculaire de la Résistance du VIH aux ARV, University of Sciences, Techniques and Technologies of Bamako; Unité d’Epidémiologie Moléculaire de la Résistance du VIH aux ARV, University of Sciences, Techniques and Technologies of Bamako; Unité d’Epidémiologie Moléculaire de la Résistance du VIH aux ARV, University of Sciences, Techniques and Technologies of Bamako; Unité d’Epidémiologie Moléculaire de la Résistance du VIH aux ARV, University of Sciences, Techniques and Technologies of Bamako; Centre Hospitalier Universitaire Gabriel Touré; Centre Hospitalier Universitaire Gabriel Touré; Northwestern University; Sorbonne Université, INSERM, Institut Pierre Louis d’Épidémiologie et de Santé Publique (iPLESP), Assistance Publique-Hôpitaux de Paris (AP-HP), Hôpital Pitié-Salpêtrière; Sorbonne Université, INSERM, Institut Pierre Louis d’Épidémiologie et de Santé Publique (iPLESP), Assistance Publique-Hôpitaux de Paris (AP-HP), Hôpital Pitié-Salpêtrière; Centre Hospitalier Universitaire Gabriel Touré; Sorbonne Université, INSERM, Institut Pierre Louis d’Épidémiologie et de Santé Publique (iPLESP), Assistance Publique-Hôpitaux de Paris (AP-HP), Hôpital Pitié-Salpêtrière

**Keywords:** SARS-CoV-2, Children, Seroprevalence, IgG, Mali, Africa, Seroprevalence, Anti-nucleocapsid antibodies

## Abstract

**Background:**

This study aimed to assess the seroprevalence of SARS-CoV-2 among children attending pediatric consultations in Bamako, Mali, using a rapid diagnostic test (RDT) on fingertip or venous blood samples.

**Methods:**

A single-center, prospective cross-sectional study was conducted from May to September 2022 at the Pediatric Hospital in Bamako, Mali. Children aged 1 to 15 years underwent phlebotomy or fingertip blood sampling for SARS-CoV-2 antibody testing using the Abbott Panbio COVID-19 IgG/IgM Test. Demographic data and potential risk factors were collected. Categorical variables were compared using Fisher’s exact test, and quantitative variables were analyzed using the Mann-Whitney test.

**Results:**

A total of 315 children were included, with a median age of 6 years (range 3–9 years); 45.7% (144/315) were younger than 6 years, and 54% (170/315) were male. The majority lived in urban areas (89.9%) and used public transportation (85.7%). The overall seroprevalence was 63.5%, with a higher seroprevalence observed among children aged 6 years and older compared to those under 6 years. The odds of having a positive serology were approximately twice as high in children aged ≥6 years in both univariate (OR 1.99; 95% CI: 1.25–3.17; P=0.0014) and multivariable analyses (OR 2.05; 95% CI: 1.26–3.32; P=0.0038). No significant differences in seropositivity were found for other demographic or risk factors.

**Conclusions:**

A substantial proportion of children in Bamako showed evidence of past SARS-CoV-2 infection, underscoring the importance of continued surveillance and preventive measures in this population.

## Introduction

The COVID-19 pandemic, which began in China in late December 2019, has already affected more than 700 million people worldwide and nearly caused 7 million deaths. With 33,000 reported cases, Mali appears to be among the least affected countries ^1^.

These data are underestimated since the diagnostic strategies by PCR tests of nasopharyngeal samples, which is the gold standard, do not make it possible to identify all the cases of infections related to SARS-CoV-2, virus responsible for COVID-19^2^. This is especially true since most African countries do not have the resources to perform this diagnosis of COVID-19 on a large scale ^3^. The ratio of estimated actual to reported infections ranged from 18:1 in South Africa to 954:1 in Nigeria, indicating high heterogeneity between countries ^4^. A seroprevalence study among healthcare workers in Bamako referral hospitals revealed a rate of 51.8%, suggesting a potentially higher overall infection rate in the community^5^

Nasopharyngeal screening tests detect the presence of the virus during an infection. They confirm the diagnosis of Covid-19. But a negative test does not provide information on past contact with the virus: it may indicate a cured infection as well as no contact with the virus. A more reliable method to evaluate the overall picture is the detection of SARS-CoV-2 antibodies, which gives more information about previous contact with the SARS-CoV-2 virus up to several months after the contact.

A meta-analysis study of SARS-CoV-2 seroprevalence in children worldwide found significant variation across waves of the pandemic, ranging from 7.39% (5.85–9.90%) in the first wave to 37.60% (18.14–59.42%) in the fifth wave and 56.69% (52.83–60.50%) in the sixth wave with significant variation across WHO regions^6^.

In Mali, a seroprevalence surveillance found an evidence of increasing exposure over time among children under 10 years ^7^.

In this context, the main purpose of this work was to estimate the seroprevalence SARS-CoV-2 antibodies in children in Bamako, Mali, three months following the country’s fourth and most significant COVID-19 wave (November 2021 to January 2022).^8^

## Methods

### Study design

This is a single-center prospective cross-sectional study to determine the seroprevalence of SARS-CoV-2 in children.

### Settings

The study recruited participants from May to September 2022 at Bamako’s Pediatric Hospital, the leading children’s medical center in Mali, treating over 30,000 young patients annually. Mali’s capital, Bamako, a diverse city of over 3 million people, is located on the banks of the Niger River. Its rapid growth is fueled by a constant influx of young migrants from other regions of Mali.

### Participants

Participants in the study were children between the ages of 1 and 15 years old (included) presenting to the pediatric outpatient clinic of the Gabriel Touré University Hospital in Bamako who had a representative of legal age who agreed to give free and informed written consent.

### Informed consent

Informed consent was obtained for each child, and assent given by children when possible. Participants were free to decline/withdraw consent at any time without providing a reason and without being subject to any resulting detriment.

### Samples and data collection

All children underwent phlebotomy or fingertip blood sampling. Samples were collected by trained biologists and pediatric nurses. Whole blood was tested for antibodies to SARS-CoV-2 core (IgM, IgG) with the Abbott Panbio COVID-19 IgG/IgM Test. This test has a sensitivity of 97.8% [95% CI 92.1%-99.7%] and a specificity of 92.8% [95% CI 88.9%-95.7%] ^9^.

Study data were collected on a case report form (CRF). Participants and their parents provided information at enrolment relating to age, gender and potential factors associated to SARS-CoV-2 seropositivity including place of residence, type of household, mode of transport, schooling of parents, number of children, number of persons living in the same household, COVID-19 contamination of parents, COVID-19 vaccination of parents, contact of parents with positive cases, notion of travel of parents

### Outcomes

First, we assessed the prevalence of antibodies anti-SARS-CoV-2 IgG in children presenting to pediatric consultations in Bamako, Mali.

Second, we estimated the proportion of symptomatic children among those with positive serology (IgG).

### Sample size

Given the constraints in the number of tests available, we anticipate that between 350 and 450 TRODS will be performed. We assume a difference in proportions of +/− 5% compared with 66%, the seroprevalence found in Africa in this meta-analysis among children worldwide^6^. With a sample size between 350 and 450, a two-sided binomial test at an alpha level of 0.05, the power will be ranging from 45% to 60%. The power calculation was made using STATA^®^, with the one-sample binomial test module (version 13).

### Statistical analysis

Demographic characteristics were described by their means, standard deviations, medians, IQR, minimums and maximums for quantitative variables. For qualitative variables, numbers and percentages by class or modality were given. The number and percentage of participants testing positive by serological screening were given for the total population. The 95% confidence intervals were calculated.

The Fisher exact test was used to compare categorical variables, and the Mann Whitney test was used to compare quantitative variables between groups.

Potential factors associated to a positive serology (IgG) were assessed among the following baseline characteristics: age, gender, place of residence, type of household, mode of transport, schooling of parents, number of children, number of persons living in the same household, COVID-19 contamination of parents, COVID-19 vaccination of parents, contact of parents with positive cases, notion of travel of parents. Univariate logistic regressions were performed to look for potential factors associated to SARS-CoV-2 positive serology. All variables tested providing a p-value of 0.10 in univariate analysis were retained for the construction of the final multivariate model.

## Results

From May 2022 to September approximatively 8723 children visited the Pediatric Hospital and we included 315 of them. The median age was 6 years (3–9 years) with 144/315 (45.7%) who were younger than 6 years (school age); 170/315 (54%) were male. The majority lived in urban areas and used public transportation to get around, 283/315 (89.9%) and 270/315 (85.7%) respectively. Most children lived in a monogamous household 223/315 (70%). Almost half of the parents were illiterate 154/315 (48.9%) and 45/315 (14.3%) received higher education. The median number of children per household was 45 (2–5) and the median number of persons living in the same household was 15 (8–20). None of the parents reported being infected with COVID-19 and 8/315 (2.5%) were vaccinated. Three parents reported had contact with positive cases Table 1

Out of 315 participants, 200 (63.5%) had positive antibodies against the SARS-CoV-2 nucleocapsid protein, ensuring that no vaccine-induced anti-spike antibodies could interfere with the result. The seroprevalence was 63.49% (95% CI: 57.91–68.82) in the study population. The proportion of COVID-19 symptomatic children among those with IgG positive serology was 93.00% (95% CI: 88.53–96.12). Among children younger than 6 years old, the seroprevalence was 54.9%, while it was 70.8% in children aged 6 years or older. In the study population, 94 out of 145 girls (64.8%) were seropositive. Among the 170 boys, 106 (62.4%) were seropositive. Therefore, 94/200(47.0%) of the seropositive children were girls. In urban areas, 63.4% of the children were seropositive (180/284), while in rural areas, the seroprevalence was 64.5% (20/31). The three children whose parents reported contact with an infected individual were all found to be seropositive. There were no positive SARS-CoV-2 cases among the parents. Every child of the 8 vaccinated parents had a positive antibody test. Children living in middle-sized households (10 to 20 people) had a similar seroprevalence (66.4%) to those living in smaller households (less than 10 people, 65.5%) and both were higher than those living in larger households (more than 20 people, 59.3%). Among those using public transportation, the seroprevalence was 62.2% (168/270), and among those using personal transportation, it was 71.1% (32/45). Children from polygamous households had a seroprevalence of 61.6%, compared to 64.2% for those from monogamous households. There was a variation in seroprevalence based on parental education level from 58.9% for koranic education to 69.2% for primary education. Seroprevalence increased progressively with increasing family size, from 60.8% for families with fewer than three children to 63.6% for those with 3 to 5 children, reaching 65.6% for those with more than 5 children.

Compared to children aged <6 years old, the proportion of positive serology was 2 times higher among those aged ≥6 years old in both univariate and multivariable analyses (OR 1.99; 95% CI: 1.25–3.17; P=0.0014) and (OR 2.05; 95% CI: 1.26–3.32; P=0.0038), respectively. There was no significant difference in proportion of SARS-CoV-2 seropositivity with other factors Table 2.

## Discussion

The seroprevalence of SARS-CoV-2 antibodies among the study population of 315 children from urban and rural areas provides valuable insights into the spread of the virus in a young, predominantly urban demographic. The overall seroprevalence was very high 63.49% (95% CI: 57.91–68.82), with differences observed across age groups, household sizes, and transportation modes. This study highlights the significant exposure of children to SARS-CoV-2, even in the absence of reported COVID-19 cases among parents and provides a snapshot of the factors influencing seropositivity in this population.

### Age-Related Seroprevalence and Potential Implications:

A key finding of the study is the higher seroprevalence observed in children aged 6 years and older (70.8%) compared to those younger than 6 years (54.9%). This age-related difference in seropositivity is statistically significant and suggests that older children may be more exposed to SARS-CoV-2, possibly due to increased mobility, social interaction, or school attendance. Previous studies have shown that school-aged children, while less likely to develop severe COVID-19 symptoms, play a crucial role in the transmission of the virus within communities^10^. The association between age and seropositivity underscores the importance of monitoring and managing SARS-CoV-2 exposure in older children, particularly as schools and public spaces continue to function during pandemic waves.

### Household Size and Seroprevalence:

The data indicates a higher seroprevalence in children living in middle-sized households (10 to 20 people, 66.4%) compared to those in smaller or larger households. This finding suggests that household size plays a role in the spread of SARS-CoV-2 among children. Middle-sized households may have more frequent intra-household interactions and shared spaces, increasing the likelihood of transmission compared to larger households, where overcrowding might paradoxically limit close contact due to the distribution of interactions among more individuals. This observation is consistent with previous research indicating that household transmission is a significant factor in the spread of SARS-CoV-2, particularly in settings where multiple family members share living spaces^11^.

### Urban vs. Rural Seroprevalence and Public Transportation:

The slight difference in seroprevalence between children from urban (63.4%) and rural (64.5%) areas suggests that the risk of exposure to SARS-CoV-2 is relatively uniform across different settings, despite the common perception that urban areas, with their higher population density, might have a higher risk. The use of public transportation, a factor commonly associated with increased exposure to respiratory infections, showed a lower seroprevalence (62.2%) compared to those using personal transportation (71.1%). This counterintuitive finding could be attributed to the specific behaviors and preventive measures adopted by families who can afford personal transportation, possibly leading to a lower perceived need for other protective measures in social settings. Alternatively, it may reflect the reduced opportunity for exposure among public transport users due to adherence to preventive measures such as mask-wearing and physical distancing, which were heavily promoted during the study period^12,13^.

### Limitations and Future Directions:

While the study provides important insights into SARS-CoV-2 seroprevalence among children, some limitations should be considered. The study’s cross-sectional design limits the ability to establish causality between observed factors and seroprevalence. Moreover, the reliance on self-reported data for parental COVID-19 status and exposure history may introduce reporting bias also Nucleocapsid-specific antibodies often disappear within a year, likely leading to a significant underestimation of past infections^14^. Future longitudinal studies could provide more definitive evidence on the factors influencing seroprevalence and help to identify specific interventions to reduce transmission among children. The study’s findings also raise questions about the role of asymptomatic and subclinical infections in the spread of SARS-CoV-2 among children. Given the high seroprevalence despite the absence of reported cases among parents, it is possible that children play a more significant role in the silent transmission of the virus than previously thought. This underscores the need for ongoing surveillance and research to understand the full impact of COVID-19 on different age groups and to inform public health strategies that protect both children and the broader community.

## Conclusions

This study highlights a high seroprevalence of anti-SARS-CoV-2 antibodies among children in Bamako, reflecting significant exposure to the virus in this young population. The results reveal significant variations based on age, household size, and mode of transportation, suggesting complex dynamics in the transmission of the virus within households and communities. Despite the high seroprevalence, the absence of reported cases among parents underscores the possibility of silent transmission, particularly among older children.

These findings underscore the importance of continuous seroprevalence monitoring among children to better understand their role in the dynamics of COVID-19 transmission. They also call for tailored public health strategies to limit the spread of the virus, particularly in schools and households. Future longitudinal studies could provide more robust data to inform necessary interventions for protecting children and reducing community transmission

## Figures and Tables

**Table 1:** Description of the sociodemographic characteristics of the participants (children and parents)

Characteristics	Overall N=315		IgG serostatus N=315				P-value*
			Negative n=115		Positive n=200		
**Age, year, median (IQR) (min - max)**	6 (3–9)	(1–15)	5 (3–8)	(1–15)	7 (4–10)	(2–15)	**0.0007**
<6	144 (45.7)		65 (56.5)		79 (39.5)		**0.0047**
≥6	171 (54.3)		50 (43.5)		121 (60.5)		
**Gender, n (%)**							0.7248
Male	170 (54.0)		64 (55.7)		106 (53.0)		
Female	145 (46.0)		51 (44.3)		94 (47.0)		
**Place of residence, n (%)**							1.0000
Urban	283 (89.9)		104 (90.4)		179 (89.5)		
Rural	31 (9.8)		11 (9.6)		20 (10.0)		
Foreign country	1 (0.3)		0 (0.0)		1 (0.5)		
**Type of transport, n (%)**							0.3160
Public	270 (85.7)		102 (88.7)		168 (84.0)		
Personal	45 (14.3)		13 (11.3)		32 (16.0)		
**Marital status of parents, n (%)**							0.6948
Monogamous	223 (70.8)		82 (71.3)		141 (70.5)		
Polygamous	86 (27.3)		33 (28.7)		53 (26.5)		
Divorced/Widowed/Single	6 (1.9)		0 (0.0)		6 (3.0)		
**Education level of parents**							0.9529
None	154 (48.9)		55 (47.8)		99 (49.5)		
Koranic	56 (17.8)		23 (20.0)		33 (16.5)		
Primary	13 (4.1)		4 (3.5)		9 (4.5)		
Secondary	47 (14.9)		17 (14.8)		30 (15.0)		
Higher	45 (14.3)		16 (13.9)		29 (14.5)		
**Number of children of parents, median (IQR) (min - max)**	4 (2–5)	(1–12)	3 (2–5)	(1–12)	4 (3–5)	(1–12)	0.3383
<3	79 (25.1)		31 (27.0)		48 (24.0)		0.8092
3–5	140 (44.4)		51 (44.3)		89 (44.5)		
≥5	96 (30.5)		33 (28.7)		63 (31.5)		
**Individuals the household, median (IQR) (min - max)**	15 (8–20)	(3–36)	15 (8–20)	(3–32)	12 (8–20)	(3–36)	0.3805
<10	87 (27.6)		30 (26.1)		57 (28.5)		0.5043
10–20	110 (34.9)		37 (32.2)		73 (36.5)		
≥20	118 (37.5)		48 (41.7)		70 (35.0)		
**COVID-19 infection status of parents, n (%)**							na
No	315 (100)		115 (100)		200 (100)		
Yes	0 (0.0)		0 (0.0)		0 (0.0)		
**COVID-19 vaccination status of parents, n (%)**							**0.0293**
No	307 (97.5)		115 (100)		192 (96.0)		
Yes	8 (2.5)		0 (0.0)		8 (4.0)		
**COVID-19 positive contact with parents**							0.5565
No	312 (99.1)		115 (100)		197 (98.5)		
Yes	3 (0.9)		0 (0.0)		3 (1.5)		
**Travelling of parents**							0.7106
No	280 (88.9)		101 (87.8)		179 (89.5)		
Yes	35 (11.1)		14 (12.2)		21 (10.5)		

na, not applicable

* P-values were obtained from Fischer’s exact test for categorical variables and from Mann-Whitney test for numerical variables

**Table 2:** Factors associated to the seroprevalence (IgG) of SARS-CoV-2

Characteristics	IgG serostatus					
	N=315					
	Negative n=115	Positive n=200	Univariable OR (95% CI)	P-value*	Multivariable OR (95% CI)	P-value*
**Age, year, median (IQR)**	5 (3–8)	7 (4–10)	1.11 (1.04–1.19)	**0.0014**		
<6	65 (45.1)	79 (54.9)	1	**0.0037**	1	**0.0038**
≥6	50 (29.2)	121 (70.8)	**1.99 (1.25–3.17)**		**2.05 (1.26–3.32)**	
**Gender, n (%)**				0.6494		0.4077
Male	64 (37.6)	106 (62.4)	1		1	
Female	51 (35.2)	94 (64.8)	1.11 (0.70–1.76)		1.22 (0.76–1.97)	
**Place of residence, n (%)**				0.9013		0.9755
Urban	104 (36.6)	180 (63.4)	1		1	
Rural	11 (35.5)	20 (64.5)	1.05 (0.48–2.28)		1.01 (0.43–2.36)	
**Type of transport, n (%)**				0.2538		0.1980
Public	102 (37.8)	168 (62.2)	1		1	
Personal	13 (28.9)	32 (71.1)	1.49 (0.75–2.98)		1.62 (0.78–3.39)	
**Marital status of parents, n (%)**				0.6737		0.8557
Monogamous	82 (35.8)	147 (64.2)	1		1	
Polygamous	33 (38.4)	53 (61.6)	0.90 (0.54–1.50)		1.06 (0.56–2.00)	
**Education level of parents**				0.9454		0.9773
None	55 (35.7)	99 (64.3)	1		1	
Koranic	23 (41.1)	33 (58.9)	0.80 (0.43–1.49)		0.82 (0.42–1.62)	
Primary	4 (30.8)	9 (69.2)	1.25 (0.37–4.25)		1.12 (0.31–3.97)	
Secondary	17 (36.2)	30 (63.8)	0.98 (0.50–1.94)		1.00 (0.49–2.05)	
Higher	16 (35.6)	29 (64.4)	1.01 (0.50–2.02)		1.05 (0.48–2.29)	
**Number of children of parents, median (IQR) (min - max)**	3 (2–5)	4 (3–5)	1.06 (0.95–1.19)	0.2929		
<3	31 (39.2)	48 (60.8)	1	0.8014	1	0.9607
3–5	51 (36.4)	89 (63.6)	1.13 (0.64–1.99)		1.00 (0.54–1.84)	
>5	33 (34.4)	63 (65.6)	1.23 (0.67–2.29)		1.08 (0.54–2.14)	
**Individuals the household, median (IQR) (min - max)**	15 (8–20)	12 (8–20)	0.99 (0.96–1.02)	0.5137		
<10	30 (34.5)	57 (65.5)	1	0.4899	1	0.6227
10–20	37 (33.6)	73 (66.4)	1.04 (0.57–1.89)		1.08 (0.58–2.02)	
≥20	48 (40.7)	70 (59.3)	0.77 (0.43–1.36)		0.79 (0.40–1.58)	
**COVID-19 infection status of parents, n (%)**						
No	115 (36.5)	200 (63.5)	na	na	na	na
Yes	0 (0.0)	0 (0.0)	na	na	na	na
**COVID-19 vaccination status of parents, n (%)**						
No	115 (37.5)	192 (62.5)	na	na	na	na
Yes	0 (0.0)	8 (100)	na	na	na	na
**COVID-19 positive contact with parents**						
No	115 (36.9)	197 (63.1)	na	na	na	na
Yes	0 (0.0)	3 (100)	na	na	na	na
**Travelling of parents**				0.6493		0.6261
No	101 (36.1)	179 (63.9)	1		1	
Yes	14 (40.0)	21 (60.0)	0.85 (0.41–1.74)		0.83 (0.38–1.78)	

na, not applicable

* P-values were obtained from logistic regression.

## Data Availability

The data and materials generated and analyzed during this study are available from the corresponding author upon reasonable request. This includes the raw datasets, supplementary materials, and any additional information that may be necessary for replicating the study’s findings or for conducting further analysis. We are committed to promoting transparency and reproducibility in research, and we welcome inquiries for data sharing that align with ethical guidelines and participant confidentiality. All requests will be evaluated on a case-by-case basis to ensure compliance with any applicable legal and ethical standards.
